# The Effect and Mechanism of Transdermal Penetration Enhancement of Fu’s Cupping Therapy: New Physical Penetration Technology for Transdermal Administration with Traditional Chinese Medicine (TCM) Characteristics

**DOI:** 10.3390/molecules22040525

**Published:** 2017-03-27

**Authors:** Wei-Jie Xie, Yong-Ping Zhang, Jian Xu, Xiao-Bo Sun, Fang-Fang Yang

**Affiliations:** 1 School of Pharmacy, Guiyang College of Traditional Chinese Medicine, No. 50 Shi Dong Road, Guiyang 550002, China; xwjginseng@126.com (W.-J.X.); twt8489@126.com (J.X.); yff19771128@126.com (F.-F.Y.); 2 Institute of Medicinal Plant Development, Chinese Academy of Medical Sciences & Peking Union Medical College, Beijing 100193, China; ginseng123@163.com

**Keywords:** transdermal physical penetration technology, Fu’s cupping therapy, pharmacokinetics, scanning electron microscopy (SEM), transmission electron microscope (TEM), stratum corneum, mechanism

## Abstract

Background: In this paper, a new type of physical penetration technology for transdermal administration with traditional Chinese medicine (TCM) characteristics is presented. Fu’s cupping therapy (FCT), was established and studied using in vitro and in vivo experiments and the penetration effect and mechanism of FCT physical penetration technology was preliminarily discussed. Methods: With 1-(4-chlorobenzoyl)-5-methoxy-2-methylindole-3-ylacetic acid (indomethacin, IM) as a model drug, the establishment of high, medium, and low references was completed for the chemical permeation system via in vitro transdermal tests. Furthermore, using chemical penetration enhancers (CPEs) and iontophoresis as references, the percutaneous penetration effect of FCT for IM patches was evaluated using seven species of in vitro diffusion kinetics models and in vitro drug distribution; the IM quantitative analysis method in vivo was established using ultra-performance liquid chromatography-tandem mass spectrometry technology (UPLC-MS/MS), and pharmacokinetic parameters: area under the zero and first moment curves from 0 to last time t (AUC*_0–t_*, AUMC*_0–t_*), area under the zero and first moment curves from 0 to infinity (AUC*_0–∞_*, AUMC*_0–∞_*), maximum plasma concentration (C_max_) and mean residence time (MRT), were used as indicators to evaluate the percutaneous penetration effect of FCT in vivo. Additionally, we used the 3^K^ factorial design to study the joint synergistic penetration effect on FCT and chemical penetration enhancers. Through scanning electron microscopy (SEM) and transmission electron microscope (TEM) imaging, micro- and ultrastructural changes on the surface of the stratum corneum (SC) were observed to explore the FCT penetration mechanism. Results: In vitro and in vivo skin permeation experiments revealed that both the total cumulative percutaneous amount and in vivo percutaneous absorption amount of IM using FCT were greater than the amount using CPEs and iontophoresis. Firstly, compared with the control group, the indomethacin skin percutaneous rate of the FCT low-intensity group (FCTL) was 35.52%, and the enhancement ratio (ER) at 9 h was 1.76X, roughly equivalent to the penetration enhancing effect of the CPEs and iontophoresis. Secondly, the indomethacin percutaneous ratio of the FCT middle-intensity group (FCTM) and FCT high-intensity group (FCTH) were 47.36% and 54.58%, respectively, while the ERs at 9 h were 3.58X and 8.39X, respectively. Thirdly, pharmacokinetic data showed that in vivo indomethacin percutaneous absorption of the FCT was much higher than that of the control, that of the FCTM was slightly higher than that of the CPE, and that of the FCTM group was significantly higher than all others. Meanwhile, variance analysis indicated that the combination of the FCT penetration enhancement method and the CPE method had beneficial effects in enhancing skin penetration: the significance level of the CPE method was 0.0004, which was lower than 0.001, meaning the difference was markedly significant; the significance level of the FCT was also below 0.0001 and its difference markedly significant. The significance level of factor interaction A × B was lower than 0.0001, indicating that the difference in synergism was markedly significant. Moreover, SEM and TEM images showed that the SC surfaces of Sprague-Dawley rats treated with FCT were damaged, and it was difficult to observe the complete surface structure, with SC pores growing larger and its special “brick structure” becoming looser. This indicated that the barrier function of the skin was broken, thus revealing a potentially major route of skin penetration. Conclusion: FCT, as a new form of transdermal penetration technology, has significant penetration effects with TCM characteristics and is of high clinical value. It is worth promoting its development.

## 1. Background

Over the past several decades, much work has been done to overcome the skin barrier for drug penetration and increase the number of drugs available for transdermal administration, mainly relating to the skin´s stratum corneum (SC) [1,2,3,4,5,6]. This work mainly includes the development of chemical penetration enhancement, physical enhancement, and biochemical enhancement methods for transdermal drug delivery. Common examples are chemical penetration enhancers (CPEs) [1,7], sonophoresis [8,9], iontophoresis [1,10,11] and microneedles [3,10,11,12]. However, to date, the transdermal delivery for most drugs remains a significant challenge due to low permeation rates [10], in particular for larger molecular weight drugs.

**Figure 1:**
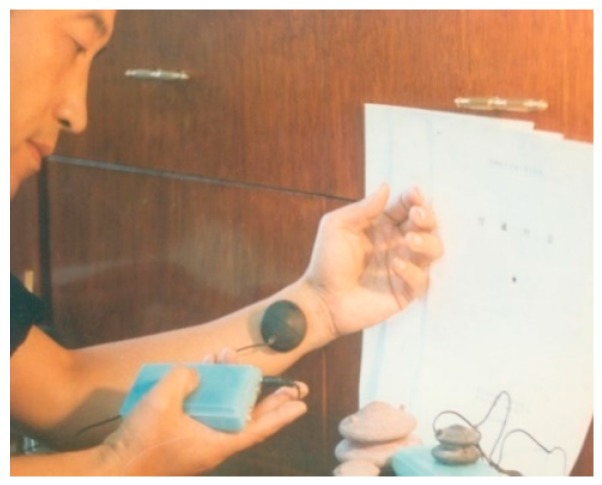
Example of Fu´s cupping therapy (FCT) treatment on human skin.

**Figure 2:**
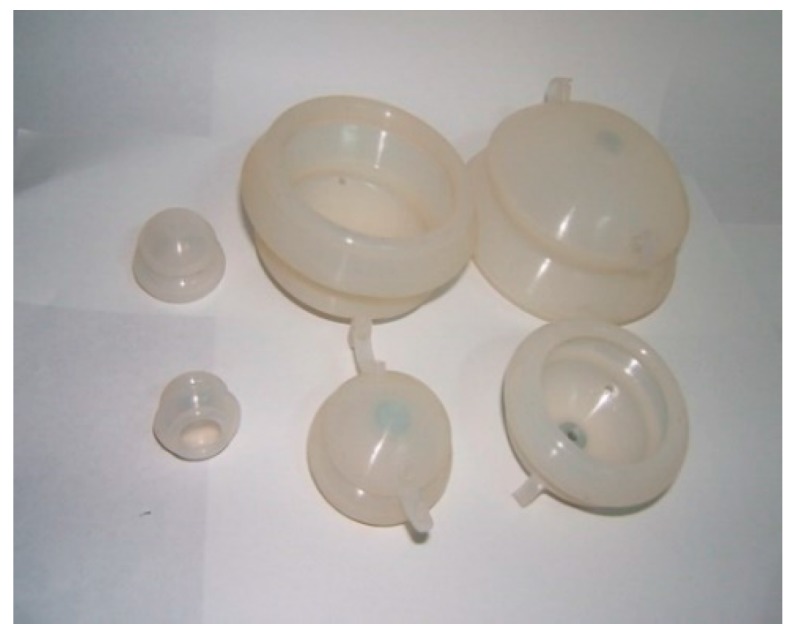
Fu’s cupping made of white rubber.

Cupping therapy is one of the five traditional Chinese medicine (TCM) therapies (which are medicine, acupuncture, moxibustion, massage, and cupping), and the earliest ancient records about cupping therapy can be traced back to *Prescriptions of Fifty-two Diseases* from the period of the Tang Dynasty, which are documents about the treatment of diseases using “horn cups” (made of ox horn). Fu’s cupping therapy (FCT) marks the comprehensive modernization, innovation, and development of TCM cupping therapy (Figure 1 and Figure 2). It encloses and creates a confined space between the body skin (or mucosa) and the walls of the cup through the FCT effect, which shapes and serves as an artificial environment producing various stimulating effects on human skin and subcutaneous structure. Multiple physical, chemical, and biological effect parameters within the cup cavity, such as pressure, temperature, and O_2_ and CO_2_ amounts, can be observed and regulated through its observation and regulation slots on the cup, respectively. Since the cup cavity is artificially constructed and presses on the body, it is referred to as “artificial near-body microenvironment full regulation therapy”, namely through the theory of “generalized meridian: 3 fields and 2 cavities–gap”, forming part of TCM theories. Furthermore, several effects of FCT as a TCM treatment on the human body, especially on the skin, have been revealed via modern research. Among them, several aspects may support and interpret the idea of FCT being used as a method of enhanced drug penetration.

Based on the above, we focused on a preliminary exploration of utilizing FCT and creating a new physical technology for transdermal administration with TCM characteristics. A 1-(4-chlorobenzoyl)-5-methoxy-2-methylindole-3-ylacetic acid (indomethacin, a model drug) hydrophilic gel patch [13,14] was prepared as preparations for transdermal administration, while naproxen acted as an internal standard. Firstly, compared with CPEs (as references, including Azone and mint oil) and iontophoresis, FCT penetration enhancement was divided into three groups (Table 1): the FCT low-intensity group (FCTL), the FCT middle-intensity group (FCTM), and the FCT high-intensity group (FCTH). Their penetration effects on indomethacin hydrophilic gel patches were evaluated through in vitro and in vivo transdermal penetration enhancement tests, in which in vitro transdermal parameters and in vivo pharmacokinetic parameters were used as detection indices. Posteriorly, the synergism between FCT physical penetration technology and chemical penetration enhancers (regarding the various CPE ratios) in transdermal enhancement was investigated first, utilizing 3^K^ factorial design and ultra-performance liquid chromatography-tandem mass spectrometry technologies (UPLC-MS/MS). Furthermore, skin treated with chemical penetration enhancers, iontophoresis, and FCT, obtained from in vitro skin penetration studies, was examined via scanning electron microscopy (SEM) [15,16] and transmission electron microscopy (TEM) [5,9,15,16,17,18,19,20] to investigate the effect of FCT physical penetration technology on ultrastructural skin changes, especially its SC, and to tentatively and preliminarily evaluate the mechanism by which FCT enhances skin penetration.

## 2. Methods

### 2.1. Materials

Indomethacin and naproxen references (purity > 99.9%, determined by high performance liquid chromatography) were obtained from the Chinese National Institutes for Food and Drug Control. HPLC-grade acetonitrile, methanol, and formic acid were obtained from Tedia Company Incorporation (Fairfield, OH, USA), and medical Azone and mint oil were purchased from Tianjin Kemiou Chemical Reagent Co., Ltd. (Tianjin, China). Double-distilled water was purified through a Milli-Q water purification system (Millipore, Billerica, MA, USA), and indomethacin hydrophilic gel patches were prepared and supplied by the Preparation Laboratory of Guiyang College of Traditional Chinese Medicine. Hydrophilic gel matrix materials, used to prepare indomethacin patches, were purchased from Shanghai Ye Source Biotechnology Co., Ltd. (Shanghai, China). Finally, Sprague-Dawley (SD) rats, weighing 320 to 350 g, were supplied by the Experimental Animal Center of Third Military Medical University (Chongqing, China).

### 2.2. Preparation of Indomethacin Patches

We identified the prescription of the indomethacin matrix by screening and optimization: its composition was 17.350 g of polyvinyl alcohol (PVA), 9.999 g of gelatin, 6.260 g of polyvinyl pyrrolidone (PVPK-30), 8.140 g of sodium carboxyl methyl cellulose (CMC-Na), 3.000 g of carbomer-940, 12.880 g of glycerol, 12.880 g of propylene glycol, and 4.200 g of triethanolamine.

The preparation process of matrix patches was as follows: firstly, PVA and gelatin weighed in prescription were properly swollen in water overnight, heated in a water bath, and then prepared as the mixed gel solution A (MGSA); secondly, PVPK-30, CMC-Na, and carbomer-940 were weighed, mixed, swollen, dissolved, heated, and prepared as the mixed gel solution B (MGSB); thirdly, after weighing and mixing glycerol, propylene glycol, and triethanolamine, model drugs were added and stirred until these drugs were completely dissolved as the mixed gel solution C (MGSB); and fourthly, the MGSB and MGSC were added to solution A correspondingly, stirred while being added, and water was added until all solutions weighed 600 g. Solutions were evenly stirred and deposited to remove air bubbles. Finally, the final MGS was quantitatively poured into a rubber ring attached to blank matrix backings (7 cm in diameter), extended and frozen for 2 h, and dried in natural conditions until a total of 30 patches were formed with the same uniform thickness and unified colors. The dosage per patch was 12.5 mg.

### 2.3. Standard Operation of FCT Penetration Enhancement

**Figure 3:**
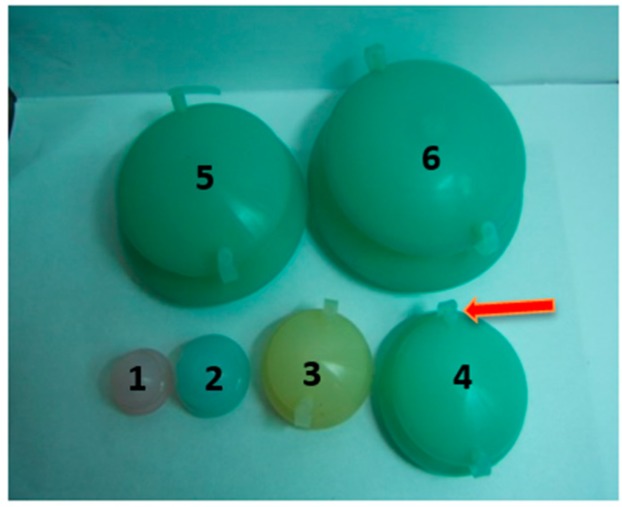
Images of cups numbering 1~6, Fu’s cupping.

**Figure 4:**
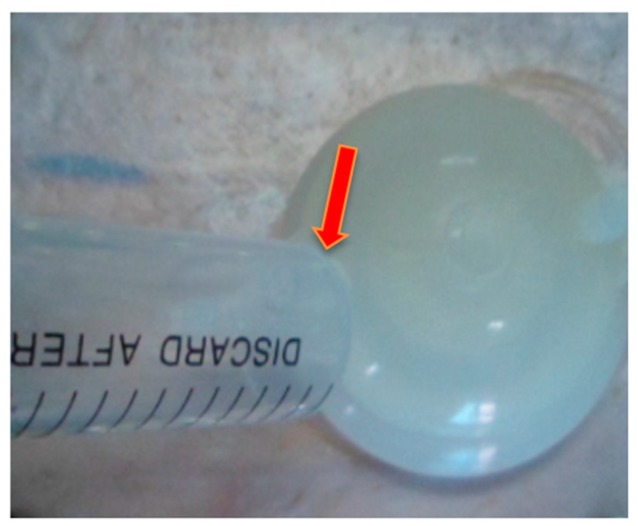
Vacuum operation of Fu’s cupping.

According to TCM theory, the FCT method influencing factors were mainly associated with the cupping method of operation, cupping therapy pressure, and duration time. Therefore, in our research, we designed and fabricated various cups of different sizes (Figure 3) to control the pressure of cupping therapy or regulate the levels of stimulation to human skin. Cup numbers 2, 3, and 4 were selected, and their inner air was evacuated using a syringe through the openings at the top of the Fu’s cupping to create and regulate the negative pressure of inner environments (Figure 3 and Figure 4).

Moreover, diverse cupping operations followed the TCM clinical operation procedure. *Retaining cupping* keeps the cup on the part to be cupped or retaining cup to follow the meridians (based on TCM theories, meridians are the body channels that contact organs and the body surface, and run Qi, blood, or body fluid; they are also defined as the trunk and branch of Qi, blood, or body fluid circulation in the human body). This is generally for 5~15 min, preferably 7 min, and with warming the meridians it is also referred to as “warming cupping”. “*Shaking cupping*” or “spinning cupping” is defined as follows: the cup on the skin is rhythmically shaken and pulled; however, the cup should be gently shaken (not too fast; once every 2 s) and at an appropriate angle. Time was measured using a stopwatch. “*Moving cupping*” also known as “slipping” or “scraping cupping”, is often applied to the larger or muscular parts of lesions. In this method, a layer of massage oil or glycerin is applied and the cup is pressed on the skin with the left hand until it is tightly secured. Then, the cup is pulled with the right hand to make it slip along the meridians, until the skin flushes.

Based on this procedure, the FCT penetration enhancement experiments were divided into three groups (Table 1).

## 3. In Vitro Transdermal Penetration Enhancement Study

### 3.1. Quantitative Methods

The Agilent 1260 HPLC system (US Agilent, Palo Alto, CA, USA,) was used for quantification with an automated variable-wavelength ultraviolet-visible detector and a C18 chromatographic column (5 μm, 250 mm× 4.6 mm, Diamonsil, Beijing, China). At chromatographic conditions, the mobile phase was composed of a mixture of acetonitrile-0.1% aqueous phosphoric acid (60:40, *v*/*v*), and the analysis was conducted at a flow rate of 1 mL/min with the detection wavelength set at 228 nm at 35 °C.

### 3.2. In Vitro Transdermal Tests

We used the Franz in vitro transdermal diffusion device [15,20] to conduct in vitro transdermal experiments [2] with the volume of receiving pool (*V*, 7.0 mL) and contact area (*A*, 2.92 cm^2^), divided into eight groups: the blank group, the 3% Azone group, the 5% Azone group, the diplex chemical penetration enhancers (DCPEs) group including 3% Azone and 5% mint oil , the iontophoresis group, and the FCT groups. Groups were processed as per the requirements of each group, and patches were used to administer drugs.

Firstly, 20% ethanol phosphate buffer saline solution (PBS) was used as the receiver solution, and the skins of SD rats were used as the percutaneous medium; the skin was tightly fit to the patches and fixed on the diffusion pool. Secondly, the receiver solution and its watery environment were completely adjusted to the constant temperature at 37 °C ± 1 °C, and electromagnetically stirred at a constant speed of 320 r/min. Finally, we immediately filled up the control receiver solution after sampling and fully withdrawing at 3 h, 6 h, 9 h, 12 h, 16 h, 24 h, 30 h, and 36 h, respectively. After such treatment, drug concentrations (*C_i_*, μg/mL) of each sample were analyzed at each time point, and the total cumulative penetration amount at all points and cumulative penetration amount per unit area (*Q_A_*, μg/cm^2^) were calculated as shown in Equation (1).

(1) $$Q_{A}=\frac{\sum_{i=1}^{n}Ci\times Vi}{A}$$

## 4. In Vivo Transdermal Penetration Enhancement

### 4.1. Quantitative Method

Analysis was performed on a WATERS Xevo TQ UPLC-MS/MS system (Waters, Milford, MA, USA), where the chromatographic column of ACQUITY UPLC Shield RP (C_18_, 1.7 μm, 2.1 mm × 50 mm, Waters) and the mobile phase of the acetonitrile-0.05% formic acid was used to enable complete separation of target components under gradient elution (Table 2). Simultaneously, the total ion chromatograms and quantitative data were recorded and processed using an electrospray ion source [21] under the following mass spectrum conditions: 150 °C ion source temperature, 3.0 KV capillary voltage, and 40 V first cone voltage. The desolventized gas (DG) was N_2_, its temperature was 550 °C, its flow was DG 750 L/h, and the cone gas flow was 50 L/h. The collision gas (CG) was Ar, and the collision gas flow rate was 0.15 mL/min. Furthermore, the indomethacin quantitative ion reaction was as follows: Parent (*m*/*z*) 356.1198→Daughter (*m*/*z*) 111.9691 + Daughter (*m*/*z*) 138.9997; meanwhile, the quantification for naproxen (an internal standard) was Parent (*m*/*z*) 231.1855→Daughter (*m*/*z*) 185.0841 (Table 3).

### 4.2. In Vivo Percutaneous Penetration Enhancement Test

SD rats were chosen and randomly divided into five groups: the control group, the FCTM group, the FCTL group, the CPE-3% Azone group, and the CPE-5% Azone group. After intraperitoneal injection and anesthesia using 10% urethane, rats were shaved and Shu-back Acupoints were marked and treated as per the corresponding group’s requirement. In addition, a work area of 3.14 cm^2^ was selected within the marked part of the skin and was used to administer drugs with dosages of 2.0 mg/rat. The 3% Azone patches were delivered to the chemical enhancer group and the none-enhancer patches were used in the control group; they were used to administer drugs after cupping therapy in the FCT group.

Capillaries with a diameter of 1.0 mm were used to take 0.5 mL of blood from the venous plexus under the rats’ eye sockets (sodium treatment was done using heparin); whole blood samples were taken at 1 h, 3 h, 6 h, 9 h, 12 h, 16 h, 20 h, 24 h, 28 h, 36 h, and 48 h, and then placed into anticoagulation *Eppendorf* tubes. Plasma was prepared by centrifugation. Then, 100 μL of rat plasma was mixed with 700 μg of internal standard, and 1 mL of ethyl acetate was added for extraction. The mixture was vortexed for 10 min and then centrifuged at 12,000 rpm for 13 min at 10 °C. Next, 800 μL of supernatant raffinate was transferred into a nitrogen blowing concentrator for evaporation into a residue. This residue was dissolved with 0.5 mL of acetonitrile, used for detection via the UPLC-MS/MS system.

## 5. Penetration Enhancement Synergism Study

The penetration enhancement synergism of the FCT (Method B) and CPE (Method A) were examined using a 3^K^ factorial design: the levels of Method A and B were divided into three grades (Table 4); nine groups of experiments were conducted, with three repeated experiments in each group for a total of 27 experiments. After detecting and analyzing with UPLC-MS/MS, the areas under the curve (AUC*_(0–t)_* and AUC*_(0–∞)_*) in the compartment model parameters and the AUMC*_(0–t)_*, AUMC*_(0–∞)_* and the maximum plasma concentration (C*_max_*)under the moment curve in the statistical moment model were used as evaluation indexes for the penetration enhancement synergistic effects, which were denoted as Y_1_, Y_2_, Y_3_, Y_4_, and Y_5_, respectively; the normalization value (Y) of all indexes was calculated according to the following formula: Y = Y_1_/Y_1max_ + Y_2_/Y_2max_ + Y_3_/Y_3max_ + Y_4_/Y_4max_ + Y_5_/Y_5max_ (Table 5).

## 6. Skin Structure Study by SEM and TEM

### 6.1. Grouping and Treatment of Animals

SD rats were randomly divided into 10 groups: the control group, the FCTL group, the FCTM group, the FCTH group, the CPE-3% Azone group, the CPE-5% Azone group, the FCTL + CPE-3% Azone group, the FCTM + CPE-5% Azone group, the FCTL + CPE-5% Azone group, and the FCTM + CPE-3% Azone group. An electric knife was used to dehair the abdomen and back skin of SD rats. After dehairing, the skin was cleaned with warm water and medical absorbent cotton was used for drying. After observing under a magnifying glass, the skin without any damage was reserved. Furthermore, the FCT groups were treated with the FCT standard operation (Table 1) for some time; in the CPE groups, patches with 3% Azone or 5% Azone were stuck to the skin and treated for 12 h; the control group was treated for 12 h using patches without any enhancers.

### 6.2. Sample Preparation and Observation

After animals were treated, skin samples were cut from the treated parts; 4% paraformaldehyde solution was firstly used to fix the sample, and then 2.5% glutaraldehyde was used to solidify them, after which they were placed in a refrigerator for preservation and to solidify at 2~4 °C for 24 h. The 2 mm × 2 mm tissue block was dehydrated, dried, and subjected to “metal spraying” plating. Then, the surface structure of skin SC was observed by a JSM-6940 SEM (Hitachi, Tokyo, Japan). After the 1 mm × 1 mm tissue block was rinsed, dehydrated, soaked, embedded, and treated in other methods using 0.1 mM/mL phosphate buffered solution, the ultra-thin sections were prepared and stained, and the SC ultrastructures were observed with a Hitachi-7650 TEM (Hitachi).

## 7. Results

### 7.1. Analysis of In Vitro Results

**Figure 5:**
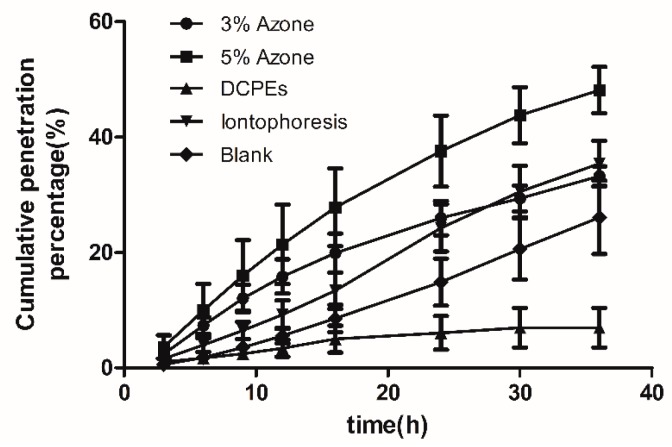
Cumulative penetration percentage (Q %)-time curve of in vitro percutaneous tests on the reference groups (*n* = 6). (●) CPE-3% Azone group; (■) CPE-5% Azone group; (▲) DCPEs group, the diplex chemical penetration enhancers group including 3% Azone combined with and 5% mint oils; (▼) iontophoresis group; (♦) blank group.

**Figure 6:**
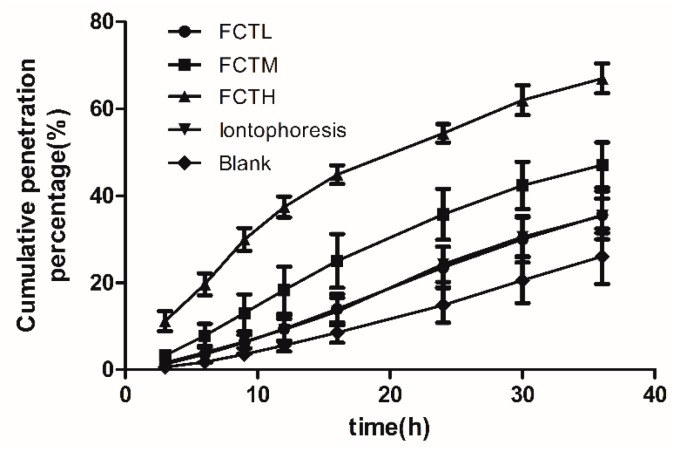
Q%-time curves of in vitro percutaneous tests on the FCT penetration enhancement groups (*n* = 6). (●) FCTL group; (■) FCTM group; (▲) FCTH group; (▼) iontophoresis group; (♦) blank group.

**Figure 7:**
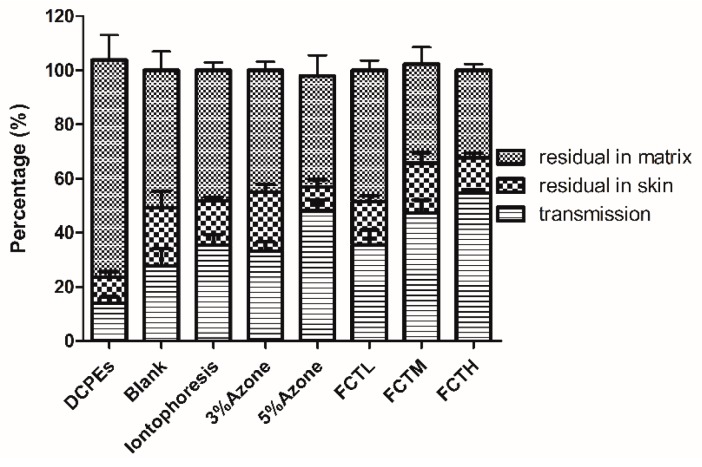
Illustrative diagram of in vitro transdermal drugs distribution. (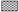) indomethacin residual in the matrix patch; (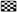) indomethacin residual in skin; (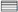) transmission indomethacin.

The ratio of cumulative transdermal penetration amount to total dosage of indomethacin, i.e., cumulative penetration percentage (Q%), was calculated according to the in vitro cumulative transdermal penetration amount [18] of indomethacin (Q, μg) at each point; and the “1 − Q%” denoted the remaining dosage percentage of the matrix at different time points. The cumulative penetration percentage (Q%) was used as the y-coordinate, and time was used as the x-coordinate to draw out the Q%-time curve of indomethacin in in vitro percutaneous diffusion tests (Figure 5 and Figure 6). Compared with the blank group, Q_A9_ and Q _A24_, and the enhancement ratio (ER), were calculated at 9 h and 24 h, as shown in Table 6. After the experiments, the matrix patch and skin were removed and cut into pieces, and then extracted for 90 min using 50 mL methanol and heating reflux. Then, the indomethacin residual in the matrix patch and skin with each extraction solution was detected to obtain the indomethacin distribution in paste, skin, and receiver solution (Figure 7). In vitro results (Figure 5 and Figure 6) showed that FCT had a significant effect on transdermal penetration enhancement. Firstly, compared with the control group, the indomethacin percutaneous ratio (Figure 7) of the FCTL group as a result of penetration enhancement was 35.52%, indicating that the FCTL group, CPE group (33.21%), and iontophoresis group (35.36%) were roughly equivalent with respect to the effects of penetration enhancement. Furthermore, the ER of the FCTL at 9 h was 1.76X that of the control group, showing a significant effect of penetration enhancement (Table 6). Secondly, the indomethacin penetration ratios (Figure 7) of the FCTM and FCTH groups were 47.36% and 54.58%, respectively, with ERs at 9 h 3.58X and 8.39X that of the control, respectively. This means that their effects on penetration enhancement were significant and higher than those of the CPEs and iontophoresis groups. Thirdly, the ERs of the FCTM and FCTH groups at 24 h were 2.35X and 1.76X, respectively (Table 6), slightly higher than that of the CPEs and iontophoresis groups; nevertheless, the decrease of penetration enhancement intensity appearing in later stages might be associated with the recovery of skin barrier function with time. Thus, the recoverability of skin barrier function after treatment by FCT must be evaluated in subsequent studies. Besides, we found that compared with the control group, the DCPE of 3% Azone + 5% mint oil reduced the IM percutaneous ratio, which was only 14.1%; however, its ER at 9 h was 0.41X, indicating an inhibitory percutaneous effect of DCPEs on indomethacin.

### 7.2. Analysis of In Vivo Pharmacokinetic Results

**Figure 8:**
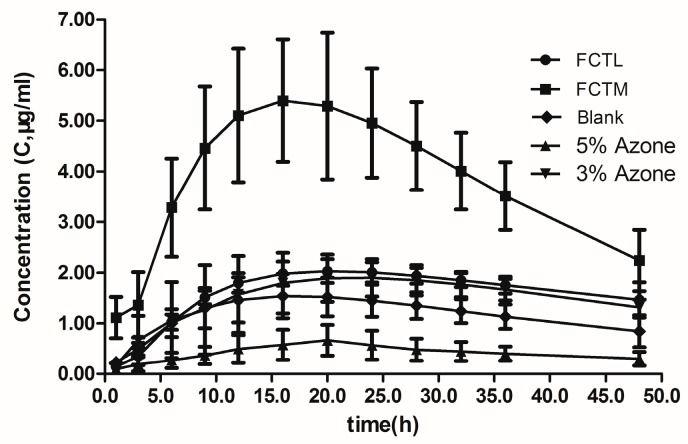
Concentration–time curve of each groups. (●) FCTL group; (■) FCTM group; (♦) Blank group; (▲) CPE-5% Azone group; (▼) CPE-3% Azone group.

According to the blood concentration of each group, the concentration (C, μg/mL)-time (t, h) curve of each group was drawn (Figure 8). The single-compartment model and statistical moment model were adopted to analyze pharmacokinetic results under concentration weight (W = 1). Analysis software Excel and DAS2.0 were combined to calculate and obtain the pharmacokinetic parameters. AUC_(0–t)_ and AUC_(0–∞)_ in the compartment model parameters and AUMC_(0–t)_, AUMC_(0–∞)_, and C _max_ in the statistical moment model were applied as in vivo evaluation indexes for comparison and analysis of penetration enhancement effects (Table 7 and Table 8).

Findings showed that the C-t curve of the FCTM group was significantly higher than that of any other group, while the C-t curve of the FCTL group was slightly higher than that of the chemical penetration enhancer group. The AUC and AUMC of in vivo percutaneous absorption of FCT penetration enhancement was higher than that of the control group, AUC and AUMC of the FCTL group were slightly higher than that of the CPE group, and the AUC and AUMC of the FCTM group were significantly higher than that of any other group, indicating that FCT had a facilitating effect on IM transdermal absorption.

### 7.3. Variance Analysis of Synergism Study

**Figure 9:**
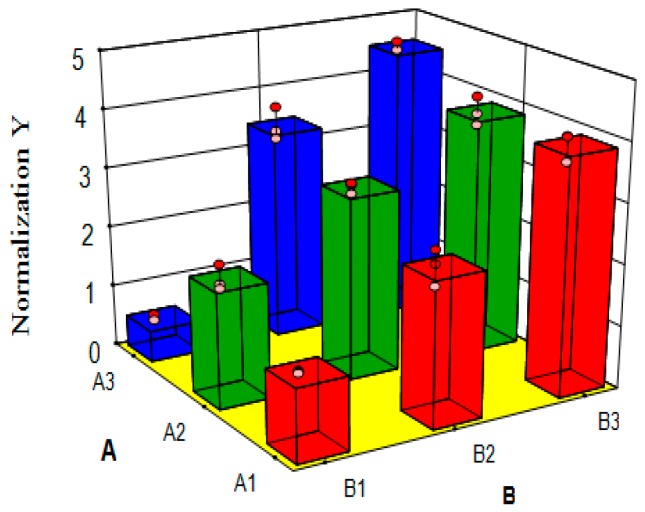
3D image of Factor A and B subjective effects: “A1→A3” and “B1→B3” mean that with the levels of Method A and B gradually increasing, the response value (Y), increases significantly.

**Figure 10:**
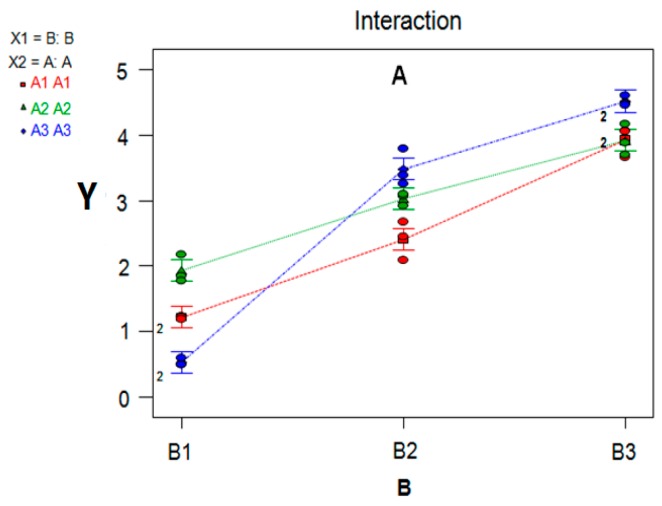
Illustrative diagram of the Factor A × B interaction: under respective conditions of A1 (the red polyline), A2 (the green polyline), and A2 (the blue polyline), Y increases significantly as the levels of Method B gradually increase (B1→B3). The polylines are not parallel, indicating a significant interaction between A and B.

Normalization values (Y) of AUC_(0–t)_, AUC_(0–∞)_, AUMC_(0–t)_, AUMC_(0–∞)_, and C_max_ were used as indices (Table 6), and Design-Expert 8.0.5 was used for regression and variance analysis. A, B and A × B interaction effects were shown by making a three-dimensional (3-D) chart analysis and visual analysis (Table 9, Figure 9 and Figure 10).

Findings were as follows: firstly, the variance analysis of regression model (*P* < 0.0001, Table 9) and the multiple correlation coefficient (R^2^ = 0.9856) suggested that this model had a significant difference, indicating that the model was good; secondly, the significance level of A (CPE) was 0.0004 and the difference was markedly significant, indicating that CPE had a markedly significant effect on the experiments; and thirdly, the significance level of B (FCT) was under 0.0001 and the difference was markedly significant, and when combined with the 3-D chart of subjective effects (Figure 9), it had a significant effect on the in vivo percutaneous absorption of indomethacin. Besides, the significance level of A × B interaction was beneath 0.0001 and its difference was markedly significant (Table 9), indicating that A × B interaction had a markedly significant effect on the in vivo percutaneous absorption of IM. Combined with the A × B interaction visual chart (Figure 10), this also demonstrated that the combination of FCT and CPEs generated a significant synergistic effect on the penetration enhancement of indomethacin.

### 7.4. Analysis of SEM Images

**Figure 11:**
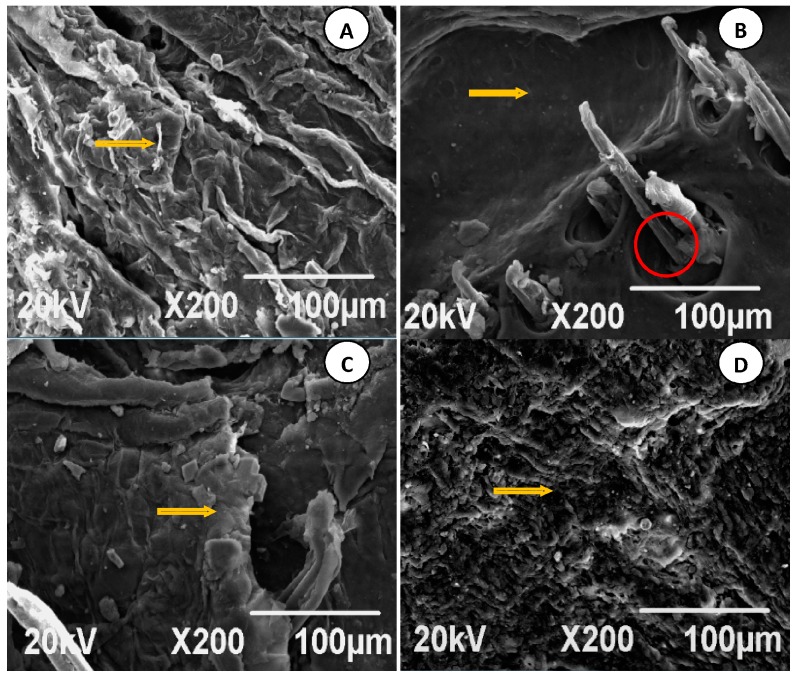

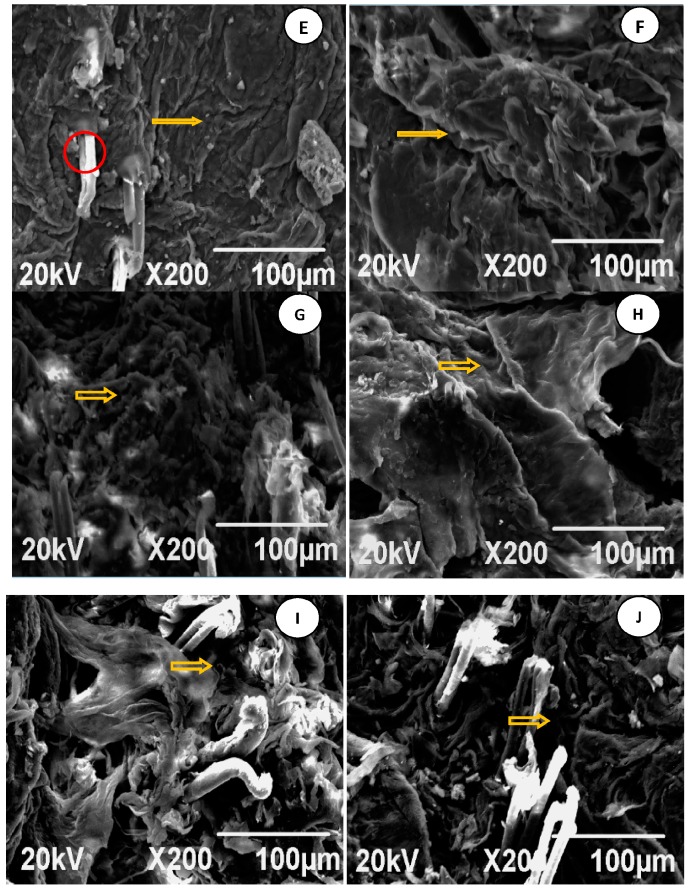
Scanning electron microscopy (SEM) images (400 × 300 pixels with ×200X resolution) of Sprague-Dawley (SD) rats skin stratum corneum (SC) in each group after the penetration enhancement treatment, were reduced to a size of 60.0 mm × 70.0 mm. (**A**) control group; (**B**) FCTL group; (**C**) FCTM group; (**D**) FCTH group; (**E**) CPE-3% Azone group; (**F**) CPE-5% Azone group; (**G**) FCTL + CPE-3% Azone group; (**H**) FCTM + CPE-5% Azone group; (**I**) FCTL + CPE-5% Azone group; (**J**) FCTM + CPE-3% Azone group.

SEM images are shown in Figure 11A–J. Control group images indicated that the SC surface cells of SD rats were flat, relatively regular, and closely spaced with some ridgelines and zigzagging arrangement, as shown in the yellow-arrow part (Figure 11A).

In comparison with the control group (Figure 11A), the FCTL (Figure 11B) and FCTM (Figure 11C) images had more cracks, and loose and irregular arrangement on the SC surface structure; meanwhile, the SC surface presented some microstructure slices, and skin SC pores grew bigger as indicated by the red circle. Furthermore, with the increase of FCT penetration enhancement, the SC structure fissure grew bigger, as marked by the yellow arrows. The SC surface of SD rats in the FCTH group (Figure 11D) was almost completely damaged, and it was hard to observe the complete SC surface structure. This revealed that the dense structure of the SC had been damaged to varying degrees by diverse FCTs, which was probably part of the percutaneous penetration mechanism.

Compared with the control group (Figure 11A), the CPE-3% Azone group (Figure 11E) and CPE-5% Azone group (Figure 11F) had slightly bumpy wrinkles, and relatively loose and irregular arrangement on the flat surface structure of the SC. Additionally, with the increase of CPE penetration enhancement, the SC grew bigger (as marked by the yellow arrows).

In contrast to the FCT groups, the SC surface of SD rats treated by CPEs had internal bumpy wrinkles, clearly shown in Figure 11E,F. There were no significant cracks, and relatively loose arrangement, but less than in the FCT groups; besides, the SC surface pores did not grow bigger (as marked by the red circle).

Compared with the control group (Figure 11A), the skin in the FCT + CPE group after treatment (Figure 11G–J) showed obvious folds and ridges, and this ridge-like structure obviously increased. The SC surface was arranged more irregularly and disorderly. Additionally, the gaps and cracks of SC surface were larger than that of the single FCT group and single penetration enhancer group, indicating that the combination of FCT and chemical penetration enhancers had more significant changes in the SC.

**Figure 12:**
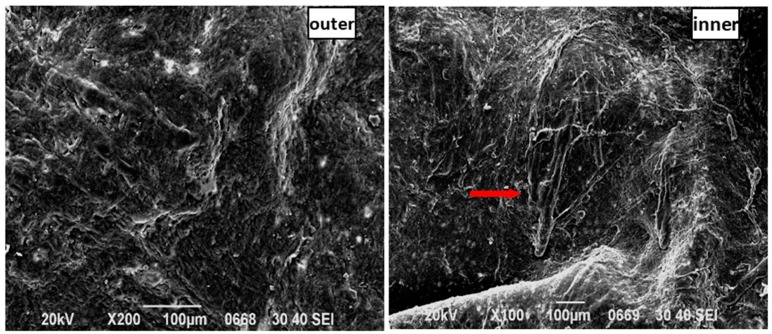
SEM images of the FCTH group: the inner and outer surfaces of SD rat skin.

Furthermore, the skin SC from the FCTH group was severely damaged, and the lower portion of the vascular structure (vessels or micrangiums) beneath the SC was observed (marked by the red arrow) (Figure 12). Several areas of skin from all the samples had cracks after 24 h during the in vitro percutaneous absorption experiments, indicating that FCTH significantly decreased the barrier effect of the skin. However, there may be self-recovery of skin barrier function after treatment, and therefore further study is needed to clarify this issue.

### 7.5. Analysis of TEM Images

**Figure 13:**
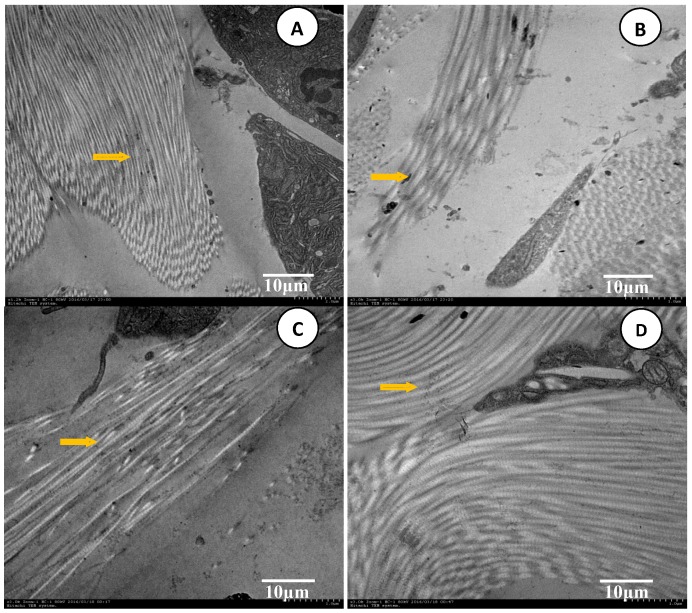
SEM images (16 × 3000X) of rat skin SC in each group after the penetration enhancement treatment were obtained from a Hitachi TEM System, and were then reduced to 70.0 mm × 67.8 mm. (**A**) control group; (**B**) FCTL group; (**C**) CPE-3% Azone group; (**D**) CPE-5% Azone group.

The control group (A), FCTL group (B), CPE-3% Azone group (C), and CPE-5% Azone group (D) are shown in Figure 13, respectively.

The control group (Figure 13A) indicated that the keratinocyte SC ultrastructure of normal SD rats had a compact “brick structure”, which was regularly and closely arranged, overlaid layer-by-layer, and had densely spaced and flat-shaped lipid composition between the layers. It constituted the complete lipid film with a compact structure, low water content, and high resistance, which has the function of barrier protection and is considered to be the major obstacle to the transdermal absorption of drugs (see yellow arrow).

Compared with the control group, after the treatment of FCT penetration enhancement as shown in Figure 13B, this “brick structure” became loose, the gap increased, and irregular arrangement and lower degree of overlaying were observed. Such changes can reduce the resistance of drug penetration and increase transdermal absorption.

Compared with the control group, the SC ultrastructure after the chemical penetration enhancer treatment (Figure 13C,D) had loose arrangement, and a reduced gap, but this change was less clear than that of the FCT group. Furthermore, the intersecting degree of the keratinized structure was significantly reduced, as indicated by the yellow arrow.

## 8. Discussion

This research attested through in vitro and in vivo percutaneous experiments, that as a new physical technology for transdermal administration, FCT can enhance IM percutaneous absorption. Meanwhile, the FCT and CPEs had a markedly synergistic effect on penetration enhancement. SEM and TEM results showed that the SC structure significantly changed after treatment, which reduced the barrier function and increased percutaneous absorption of drugs. Thus, we verified that the first action mechanism of FCT is closely interrelated to its physical effects (negative pressure). It may involve some action mechanisms of percutaneous penetration that enhance FCT physical effects as a result of subatmospheric pressure of FCT, making the fissures, keratinocyte proliferation and acidic liquid secretion more evenly scattered on skin epidermis through stimulating effects of heat, force, bioelectricity and other physical factors.

## 9. Conclusions

Therefore, FCT as a new form of physical technology for transdermal administration with TCM characteristics has a significant effect on penetration enhancement, and has high value for TCM clinical applications. Therefore, it is worth being disseminated and further developed. Its potential mechanism of penetration enhancement and FCT stimulation are closely associated with changes in the SC, and biological regulation of the skin. However, the functional mechanism of penetration enhancement must be further researched by fluorescence micro-imaging techniques, and various TCM model drugs are required to further validate the effects and features of FCT penetration enhancement.

## Figures and Tables

**Table 1:** Treatment condition parameters of FCT transdermal penetration enhancement. FCTL: FCT low-intensity group; FCTM: FCT middle-intensity group; FCTH: FCT high-intensity group.

FCT Groups	Cupping Operation	Cupping Number	Time
FCTL	Retaining cupping	2	6.0 min
FCTM	Shaking cupping	3	11.0 min
FCTH	Moving cupping	4	15.0 min

**Table 2:** Gradient change of the mobile phase.

Time	Flow Rate (mL/min)	Aqueous Phase (%)	Organic Phase (%)	Curve
0.0	0.3	64.0	36.0	6
1.0	0.3	54.0	46.0	6
2.1	0.3	48.0	52.0	6
4.5	0.3	25.0	75.0	6
5.0	0.3	10.0	90.0	6
6.0	0.3	10.0	90.0	6
6.5	0.3	64.0	36.0	6
7.5	64.0	36.0	38.0	6

**Table 3:** Mass spectrum conditions of quantitative ion peak. IM: indomethacin

Compound	Parent (*m*/*z*)	Daughter (*m*/*z*)	Dwell (s)	Cone (V)	Collision (V)
IM number 1	358.1198	138.9997	0.49	18	20
IM number 2	358.1198	111.9691	0.49	16	48
Naproxen	231.1855	185.0841	0.49	16	14

**Table 4:** Factors and levels of Method A and B. CPE: chemical penetration enhancers.

Levels	CPE (A)	FCT (B)
1	Blank (A1)	Blank (B1)
2	3% Azone (A2)	FCTL (B2)
3	5% Azone (A3)	FCTM (B3)

**Table 5:** 3^K^ factorial design experiments and results (mean, *n* = 3). Y: normalization value.

No.	Arrangement		Indexes					
	A	B	AUC*_(0–t)_*	AUC*_(0–∞)_*	AUMC*_(0–t)_*	AUMC*_(0–∞)_*	C*_max_*	Y
1	A2	B2	111.719	210.015	2838.514	13,136.654	3.805	3.073
2	A3	B2	180.273	232.491	4164.901	11,384.695	5.072	3.800
3	A3	B1	23.021	37.343	526.322	2601.184	0.783	0.594
4	A2	B3	183.711	246.637	4072.945	11,759.021	7.699	4.176
5	A1	B2	78.320	168.944	1992.035	17,113.618	2.548	2.678
6	A3	B3	213.673	287.876	4611.003	12,129.244	7.849	4.613
7	A2	B2	120.784	206.598	2982.561	9664.643	3.750	2.924
8	A3	B2	138.879	222.301	3203.711	10,611.719	4.571	3.262
9	A3	B3	216.276	265.211	4522.868	10,476.852	8.332	4.487
10	A1	B2	75.333	155.939	1955.883	14,172.779	2.655	2.452
11	A2	B1	78.905	130.606	1865.553	10,963.154	2.681	2.176
12	A3	B1	21.087	30.890	534.802	1450.696	0.782	0.497
13	A3	B1	22.260	31.663	539.604	1243.887	0.779	0.493
14	A2	B3	167.116	236.699	3990.846	11,035.755	6.723	3.891
15	A2	B3	176.351	219.040	3875.847	8132.089	6.972	3.706
16	A3	B2	139.788	233.713	3275.264	11,562.142	4.694	3.391
17	A1	B2	76.484	148.867	2014.481	8270.574	2.585	2.092
18	A2	B1	66.105	138.919	1837.867	6682.340	2.331	1.849
19	A1	B1	56.227	70.071	1479.349	2427.651	2.296	1.234
20	A1	B3	184.696	257.779	4263.475	9408.590	7.277	4.074
21	A1	B3	205.119	262.471	4028.318	10,487.270	6.175	4.066
22	A3	B3	218.340	260.470	4459.897	10,219.833	8.483	4.469
23	A2	B2	138.911	201.867	3177.125	9951.217	4.199	3.103
24	A2	B1	68.813	122.355	1846.695	6065.224	2.398	1.778
25	A1	B3	159.919	225.284	3750.857	10,929.334	5.941	3.667
26	A1	B1	55.597	70.376	1374.053	1969.218	2.621	1.221
27	A1	B1	56.397	68.021	1309.445	2121.255	2.436	1.190

**Table 6:** In vitro percutaneous test data of each group (mean ± standard deviation, *n* = 6). ER: enhancement ratio.

Group	Q_A9_ (μg/cm^2^)	Q_A24_ (μg/cm^2^)	ER_9_	ER_24_
Blank	23.72 ± 5.60	98.98 ± 12.82	1.06 ± 0.26	1.11 ± 0.30
3% Azone	70.72 ± 17.22	150.4 ± 22.86	2.98 ± 1.20	1.52 ± 0.27
5% Azone	62.97 ± 27.53	146.02 ± 34.86	2.65 ± 1.16	1.48 ± 0.35
DCPEs	9.82 ± 3.66	27.34 ± 5.35	0.36 ± 0.21	0.23 ± 0.12
Iontophoresis	42.17 ± 12.64	153.89 ± 33.99	1.78 ± 0.53	1.55 ± 0.34
FCTL	41.79 ± 17.72	156.21 ± 35.56	1.76 ± 0.82	1.58 ± 0.39
FCTM	84.88 ± 26.37	232.13 ± 35.04	3.58 ± 1.11	2.35 ± 0.35
FCTH	199.08 ± 14.48	361.54 ± 14.73	8.39 ± 0.61	3.65 ± 0.15

**Table 7:** Pharmacokinetic parameters of the single-compartment model (mean ± SD, *n* = 5).

Parameters	Units	Blank Group	FCTL Group	FCTM Group	3% Azone Group
t_1/2_	h	11.54 ± 2.90	26.98 ± 12.76	12.21 ± 1.43	21.24 ± 2.33
K_e_	1/h	0.06 ± 0.01	0.03 ± .0.01	0.06 ± 0.01	0.03 ± 0.00
V_1/F_	L/kg	4.47 ± 357.78	7900.62 ± 2567.20	0.00 ± 0.00	0.01 ± 0.00
AUC_(0–t)_	μg/mL∙h	60.66 ± 4.77	76.95 ± 3.46	202.54 ± 34.44	69.72 ± 7.37
AUC_(0–∞)_	μg/mL∙h	74.05 ± 8.66	140.01 ± 2.48	261.05 ± 41.86	128.45 ± 40.35
K_a_	1/h	0.07 ± 0.01	0.11 ± 0.04	0.07 ± 0.00	0.07 ± 0.03
t_1/2Ka_	h	9.84 ± 1.94	7.38 ± 1.27	9.47 ± 0.83	14.08 ± 2.35
T_lag_	h	1.58 ± 0.06	1.98 ± 0.65	1.55 ± 0.37	1.03 ± 0.42

**Table 8:** Pharmacokinetic parameters of statistical moment model (mean ± standard deviation, *n* = 5).

Statistical Moment	Units	Blank Group	FCTL Group	FCTM Group	3% Azone Group
AUC_(0–t)_	μg/mL∙h	60.21 ± 2.56	76.86 ± 6.07	198.72 ± 72.28	69.89 ± 10.13
AUC_(0–∞)_	μg/mL∙h	71.42 ± 9.80	133.22 ± 61.40	266.93 ± 106.45	135.23 ± 23.07
AUMC_(0–t)_	μg/mL∙h^2^	1389.21 ± 135.64	1974.49 ± 166.81	4615.58 ± 1490.33	1812.18 ± 148.18
AUMC_(0–∞)_	μg/mL∙h^2^	2299.00 ± 434.96	9511.82 ± 4546.77	11,145.18 ± 3525.72	7884.63 ± 6115.12
MRT_(0–t)_	h	23.0 ± 2.0	25.7 ± 0.6	23.5 ± 1.1	26.3 ± 2.1
MRT_(0–∞)_	h	31.5 ± 3.5	59.6 ± 17.1	40.7 ± 16.0	54.1 ± 16.5
VRT_(0–t)_	h^2^	125.6 ± 6.5	142.9 ± 18.2	141.3 ± 5.5	143.5 ± 5.5
VRT_(0–∞)_	h^2^	582.0 ± 619.7	3056.1 ± 4545.2	1111.2 ± 538.5	2671.0 ± 1042.3
t_1/2z_	h	13.8 ± 9.8	30.3 ± 13.2	17.8 ± 7.5	30.6 ± 5.9
T_max_	h	18.0 ± 5.2	28.0 ± 2.8	19.2 ± 3.4	24.7 ± 8.9
C_max_	μg/mL	2.39 ± 0.38	2.61 ± 0.31	8.00 ± 2.15	2.21 ± 0.55

**Table 9:** Variance analysis of 3K factorial design.

Source	Sum of Squares	Degree of Freedom	Mean Square	*F* Value	*p* Value	Significance
Model	43.85	8	5.48	145.05	<0.0001	***
A-A	0.95	2	0.48	12.62	0.0004	***
B-B	38.43	2	19.21	508.41	<0.0001	***
A × B	4.47	4	1.12	29.59	<0.0001	***
Pure error	0.68	18	0.04			
Cor total	44.53	26				

Note: **** *p* < 0.001 markedly significant difference.
